# Synthesis, Characterization and Hexavalent Chromium Adsorption Characteristics of Aluminum- and Sucrose-Incorporated Tobermorite

**DOI:** 10.3390/ma10060597

**Published:** 2017-05-30

**Authors:** Zhiguang Zhao, Jiangxiong Wei, Fangxian Li, Xiaoling Qu, Liang Shi, Haidong Zhang, Qijun Yu

**Affiliations:** 1 School of Materials Science and Engineering, South China University of Technology, Guangzhou 510640, China; zhao_zhi_guang@126.com (Z.Z.); zhsy_sgu@163.com (L.S.); 13954137511@163.com (H.Z.); concyuq@scut.edu.cn (Q.Y.); 2 Guangdong Low Carbon Technologies Engineering Centre for Building Materials, South China University of Technology, Guangzhou 510640, China; 3 School of Civil Engineering, Shaoguan University, Shaoguan 512005, China; qxl245970988@126.com

**Keywords:** tobermorite, aluminum, sucrose, adsorption, hexavalent chromium

## Abstract

Tobermorites were synthesized from the lime-quartz slurries with incorporations of aluminum and sucrose under hydrothermal conditions, and then used for adsorption of Cr(VI). The chemical components, and structural and morphological properties of tobermorite were characterized by X-ray diffraction (XRD), thermogravimetric-differential scanning calorimetry (TG-DSC), Fourier transform infrared spectroscopy (FT-IR), nuclear magnetic resonance (NMR), scanning electron microscopy (SEM), X-ray photoelectron spectroscopic (XPS) and N_2_ adsorption–desorption measurements. The formation and crystallinity of tobermorite could be largely enhanced by adding 2.3 wt.% aluminum hydroxide or 13.3 wt.% sucrose. Sucrose also played a significantly positive role in increasing the surface area. The adsorption performances for Cr(VI) were tested using a batch method taking into account the effects of pH, the adsorption kinetics, and the adsorption isotherms. The adsorption capacities of the aluminum- and sucrose-incorporated tobermorites reached up to 31.65 mg/g and 28.92 mg/g, respectively. Thus, the synthesized tobermorites showed good adsorption properties for removal of Cr(VI), making this material a promising candidate for efficient bulk wastewater treatment.

## 1. Introduction

Hydrothermal products of CaO-SiO_2_-H_2_O system are highly complex and comprised of crystalline to amorphous phases, collectively denoted as C-S-H phases [1]. Among the C-S-H phases, tobermorite (Ca_5_Si_6_O_16_(OH)_2_·4H_2_O) is technologically important, not only as the main binder in autoclaved building materials, but also due to its essential roles as adsorbents and ion exchangers relating to multifarious application in catalysis and waste management [2,3,4]. Tobermorite is formed below 180 °C undergoing an phase transition path: CaO + SiO_2_→ C-S-H gel→ tobermorite, it always appears within the first 60 min, and the growth is completed within 7 h [5,6]. Three types of tobermorite exist, namely 14 Å, 11 Å and 9 Å tobermorite, with their names relating to the d-spacing of (002) in X-ray diffraction patterns depending on the numbers of water molecule per formula unit. The 11 Å tobermorite has layered structure with a central layer of calcium octahedral accompanied by two infinite silicate chains on each side [5]. The composite layers, one calcium and two silicate layers, are bound together by an interlayer containing Ca^2+^, H^+^, Na^+^, and H_2_O [5,7].

Recently, the research interest in aluminum-incorporated tobermorite becomes hot again due to the utilization of more aluminum bearing raw materials (e.g., zeolite [4], newsprint recycling residue [8] and fly ash [9]) and optimization of its ion exchange performance [3]. It is reported that Al substitutes for Si in the Q^2^ or Q^3^ sites (symbol Q represents one SiO_4_ tetrahedron and the superscript indicates the number of other Q units to which it is bonded) [5]. Black et al. [10] deduced that charge imbalance existed in the tobermorite basing on the observation of no change in Ca/(Si + Al) ratio upon Al substitution. Therefore, charge balance has to achieve via a coupled substitution: □ + OH^−^→ Ca^2+^ + O^2−^, whereby □ represents the so-called “empty” position. The most common form of “□” is Na^+^, in addition, other metal ions such as Cd^2+^, Pb^2+^,Co^2+^, Ni^2+^ and Cs^+^ can also act as charge compensator. This characteristic gives tobermorite superior adsorption performance.

Many studies have been devoted to the exchange properties of synthetic tobermorite. Coleman et al. [7,8,11] reported that Al-substituted 11 Å tobermorite exhibited cation exchange properties and was effective in the removal of Cd^2+^, Pb^2+^, and Zn^2+^ from acidified aqueous wastes. An 11 Å tobermorite substituted with Na^+^ and Al^3+^ allows ion exchange with alkalis (Cs^+^, Rb^+^, and K^+^) and alkaline earth elements (Ba^2+^, Sr^2+^, and Mg^2+^) [12,13]. To the best of our knowledge, few studies have been conducted to study the adsorption of Cr(VI) by tobermorite or Al-substituted tobermorite. It is well known that chromium contamination is a widespread environmental problem arising from chromium residues. In nature, chromium exists mainly in two oxidation states (III and VI). Cr(VI) is a more toxic and soluble specie compared to Cr(III). Among the removal technologies for Cr(VI), adsorption has attracted the most interest because of its cost-effectiveness and simplicity. Although zeolites [14], clay [15,16], fly ash [17], activated carbon [18], magnetite nanoparticles [19], carbon nanotube (CNT) [20], and chitosan [21] are frequently used as general adsorbents of inorganic and organic compounds, alternative adsorbents are needed to be developed to improve the effectiveness. Based on the special structure and excellent performance, tobermorite should have excellent potential in the safe disposal of Cr(VI).

As expected, increases in tobermorite yield and its crystallinity have a positive impact upon the adsorption property of tobermorite [7,8]. It is well known that process kinetics is mainly ruled by the temperature dependent retrograde solubility of portlandite under hydrothermal conditions. Due to a significant increase of the solubility of CaO by sucrose at room temperature, it is possible to effectively increase the concentration of Ca^2+^ ions to generate a higher amount of C-S-H with the addition of sucrose. This study gives attention to modifications of the crystallization process in the CaO-SiO_2_-H_2_O system by the addition of sucrose. Our aim is to enhance the yield and crystallinity of tobermorite by introducing admixtures and hence to improve the adsorption property of tobermorite. Structures of tobermorite were identified by using XRD, TG-DSC, FT-IR, NMR, SEM, XPS techniques and N_2_ adsorption–desorption measurements in detail. The effects of dosage of aluminum and sucrose on the material structures were investigated to optimize synthesis conditions. Furthermore, the synthesized products were applied for removal of Cr(VI) ions from water. The influences of various important parameters, viz., pH, contact time and initial concentration on the removal were investigated. In addition, kinetics and isotherms were examined concerning the adsorption of Cr(VI).

## 2. Experimental

### 2.1. Hydrothermal Synthesis

The tobermorite samples were synthesized from slurry of calcium oxide (CaO) and ground quartz powder. Fresh calcium oxide was obtained by calcining calcium carbonate (CaCO_3_, 99.0%, Fuchen Co., Ltd., Tianjin, China) at 1000 °C for 3 h, immediately after which it was stored under vacuum. The quartz powder with average particle size of around 5 μm (SiO_2_ > 99.8%) was dried in an oven at 105 °C for 24 h to remove any water. Aluminum hydroxide and sucrose of analytical purity (Fuchen Co., Ltd., Tianjin, China) were added as admixtures. Deionized water obtained by the standard purification method was used as mix water. 

Hydrothermal syntheses were carried out in a Teflon-lined steel autoclave equipped with a power driven stirrer and a heat controller. The molar ratios of initial mixtures were according to the ideal composition of tobermorite (Ca_5_Si_6_O_16_(OH)_2_·4H_2_O). The molar ratio of Ca/Si for the control sample was designed at 0.83. For the sample with Al, the molar ratio of Ca/(Si + Al) was fixed at 0.83, and the replacement molar ratio of Si by Al was calculated as molar ratio of Al/(Al + Si) which varied from 0.03, 0.06 to 0.09. The addition of sucrose was calculated as molar ratio of sucrose/CaO, marked as Su/Ca, which was selected as 0.05, 0.1 and 0.15. Dry primary mixtures were stirred with deionized water (water/solid ratio of the slurry was 50) for 30 min in advance to guarantee the starting materials uniformly distributed. Then, the slurry was hydrothermally reacted at 180 °C for 8 h, and the synthesis reaction was conducted at a stirring rate of 100 r/min. The resultant products were collected, washed five times with deionized water and dried at 80 °C overnight.

### 2.2. Characterization Methods

The phase composition of the obtained products were characterized by XRD (X′Pert Pro, PANalytical B.V., Almelo, The Netherlands) analysis with Cu Kα radiation (λ = 1.5418 Å) at a 2θ range of 5°–70° and TG-DSC (STA 449C, Netzsch, Bavaria, Germany, 10 °C/min in N_2_ atmosphere) analysis. The chemical functional groups were investigated by FT-IR (VERTEX 70, Bruker, Karlsruhe, Germany) within the wavenumber range of 4000–400 cm^−1^ using KBr as the standard. The morphology of the products was observed by SEM (Nova NanoSEM 430, FEI, Hillsboro, OR, USA) equipped with an energy dispersive X-ray analyzer (EDX). Solid-state NMR was performed to characterize the local atomic structure. NMR spectra for ^29^Si and ^27^Al were acquired at 9.4 T using a Bruker Avance 400 spectrometer (Bruker, Karlsruhe, Germany). Spectra were recorded at 79.49 MHz and a spinning rate of 5 kHz with 5 s relaxation delay for ^29^Si NMR experiments and at 104.26 MHz and a spinning rate of 12 kHz with 1 s relaxation delay for ^27^Al NMR experiments. Brunauer–Emmett–Teller (BET) specific surface areas and pore size distributions were measured with a surface area and pore size analyzer (Nova Win2, Quantachrome, Boynton Beach, FL, USA). XPS analysis was exerted to identify the surface chemical composition. The XPS spectra (Kratos, Manchester, UK) were recorded on a Kratos AXIS Ultra with a monochromatic Al X-ray source at 75 W and in the constant pass energy mode (160 eV for the survey scan, 40 eV for the high-resolution analysis).

### 2.3. Adsorption Experiments

Adsorption experiments were conducted using a batch method in stoppered conical flasks at room temperature. All batch experiments were conducted in a mechanical shaker at a speed of 150 rpm. The Cr(VI) solutions were prepared by dissolving K_2_Cr_2_O_7_ in distilled water. The initial pH values of the Cr(VI) solutions were adjusted with 0.1 M HCl or NaOH to investigate the effects of pH value (pH = 2–6) on the adsorption ability of the synthetic samples. Solutions with pH value > 6 were not used, to avoid forming the metal hydroxide precipitates. Accurate amounts of the samples (T, T-A and T-S) (0.2 g) were mixed with the Cr(VI) solutions (50 mL, 160 mg/L) at different pH values in 150 mL conical flasks and shaken for 6 h. Then the dispersions were centrifuged to separate solution from solid followed by determination of Cr(VI) concentration in solution. The concentration of Cr(VI) was determined by using a atomic absorption spectrometer (Z-2000, Hitachi, Tokyo, Japan). 

Adsorption isotherms were obtained as follows: 0.2 g of samples were in contact with 50 mL of Cr(VI)solutions with varying initial concentrations (20–240 mg/L)at a pH of 4.0 for 6 h, then centrifuged to separate solution from solid followed by determination of Cr(VI) concentration in solution, as described earlier. 

The kinetic studies were performed as follows: 0.2 g of samples were equilibrated using an initial concentration of 160 mg/L Cr(VI)at a pH of 4.0. The samples were equilibrated for different time intervals of 5, 15, 30, 60, 90, 120, 180, or 360 min, then the phases were separated, and the Cr(VI) concentration in solution was determined.

## 3. Results and Discussion

### 3.1. Synthesis of Tobermorite

**Figure 1:**
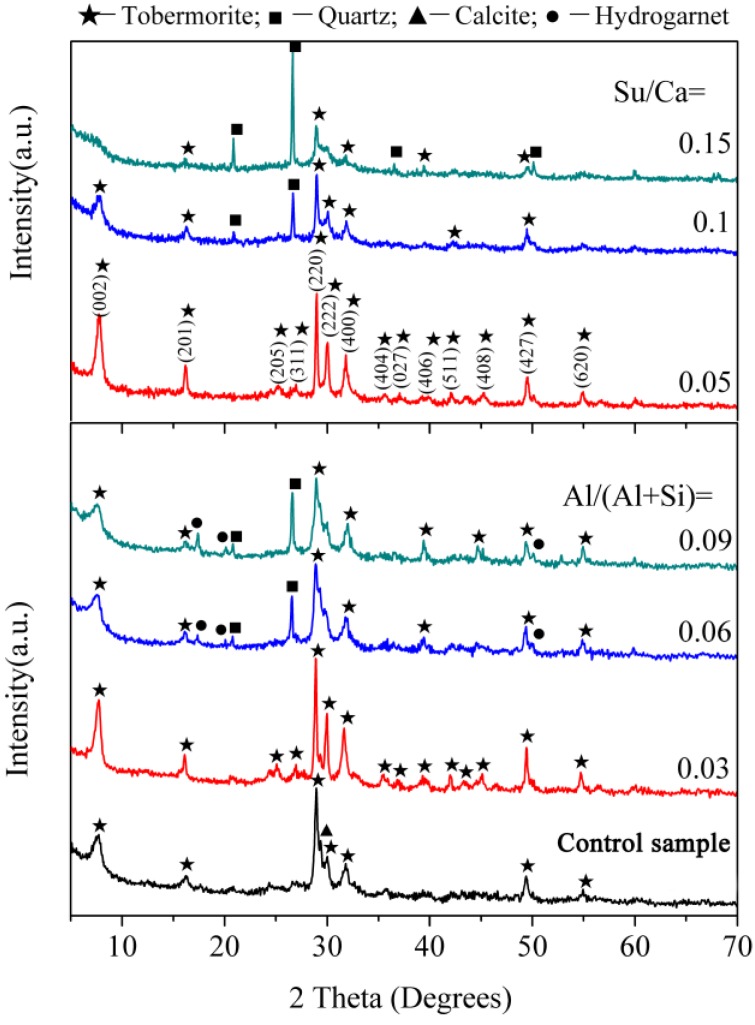
XRD patterns of the synthesized products.

XRD patterns of the synthetic samples are presented in Figure 1. The main diffraction peaks of the control sample are in good agreement with the JCPDS card (19-1364) for 11 Å tobermorite. Minor traces of calcite (CaCO_3_) with diffraction peak at 2θ = 29.3° are also present and commonly arise from atmospheric carbonation during the preparation of tobermorite. For the samples with aluminum, higher overall intensities at Al/(Al + Si) = 0.03 suggests an increased reaction rate to tobermorite. In particular, highly narrow and sharp peaks imply the high crystallinity of the tobermorite phase. The basal spacing of the (002) diffraction peak from the tobermorite phase is found to be 11.5 Å and is consistent with the previously reported values of Al-substituted 11 Å tobermorite [4]. As the aluminum addition increases, the weaker overall peak intensity of tobermorite alongside additional quartz (JCPDS card No. 70-3755) and hydro garnet (JCPDS card No. 84-1354) peaks indicate a lower reaction degree. Ease of tobermorite crystallization is known to be affected by the precursor C-S-H [2,22]. Aluminum may enhance the formation of precursor C-S-H due to lower binding energy of Al-O than that of Si-O [10] thus prompts the transformation of C-S-H to tobermorite. In the presence of excess aluminum, hydro garnet is significantly formed which retards the formation of tobermorite from the precursor C-S-H due to a lack of available Ca^2+^ in sufficient quantity [23].

For the samples with sucrose, the higher overall peak intensity of tobermorite at Su/Ca = 0.05 indicates an increased reaction rate to tobermorite. At a sucrose concentration of Su/Ca = 0.1 the weaker overall peak intensity of tobermorite alongside additional quartz peaks demonstrate a lower reaction degree. As the sucrose concentration further increases, the diffraction peaks corresponding to tobermorite become broad and ambiguous at Su/Ca = 0.15 suggesting a low crystallinity. This phenomenon may be explained that the concentration of Ca^2+^ is increased by sucrose [24]. As Al-Wakeel [25] pointed out, the concentration of Ca^2+^ has a great impact on the nucleation and growth of C-S-H. At a high level of sucrose concentration, the availability of Ca^2+^ is probably reduced due to excessively complexed calcium by sucrose [26], which retards the hydrothermal reaction.

According to the above, the purer synthesized tobermorites were prepared with aluminum addition of Al/(Al + Si) = 0.03 or at a sucrose concentration of Su/Ca = 0.05. Therefore, the above two samples of tobermorites, denoted by T-A and T-S, respectively, were preferably synthesized to conduct further studies. In addition, the control sample is marked as T to clearly explain the effects of aluminum and sucrose.

### 3.2. Effects of Aluminum and Sucrose on Tobermorite

#### 3.2.1. TG-DSC Analysis

**Figure 2:**
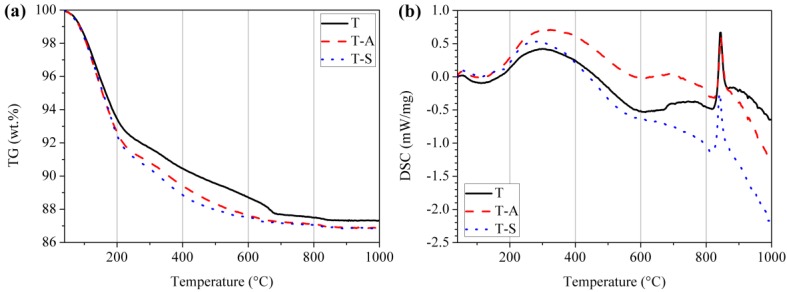
Thermogravimetry (**a**); and differential scanning calorimetry (**b**) curves of the tobermorite samples.

TG-DSC curves of the tobermorite samples are shown in Figure 2. Irrespective of the water content a mild endothermic peak occurs between 40 °C and 200 °C which corresponds with the removal of ~10 wt.% of adsorbed water and labile inter-layer water molecules of tobermorite. An exothermic peak around 840 °C is ascribed to recrystallization of tobermorite into wollastonite (β-CaSiO_3_) [25,27,28]. The weight loss of samples at each stage and the total weight loss are summarized in Table 1. Three stages of weight loss are interpreted as:

(a) 40 to 100 °C, weight loss of free water contained in tobermorite;
(b) 100 to 400 °C, weight loss of dehydration from the transformation from 11 Å tobermorite to 9 Å tobermorite [28]; and
(c) 650 to 720 °C, weight loss of decomposition of calcite [27].

It can be observed that the total weight loss of the investigated samples is in the range of 12.7–13.2% and is in the typical range for tobermorite material. Shaw et al. [5] determined a total weight loss of 12.2% for 11 Å tobermorite, which is close to our results and verifies the XRD results. The weight loss in the temperature range of 100–400 °C for the T-A sample and T-S sample is higher than that of the control sample indicates more interlayer water. Thus, there is more Ca^2+^ in the interlayer, which may be favorable for the ion exchange process when tobermorite is directly used as an adsorbent.

#### 3.2.2. FT-IR Analysis

**Figure 3:**
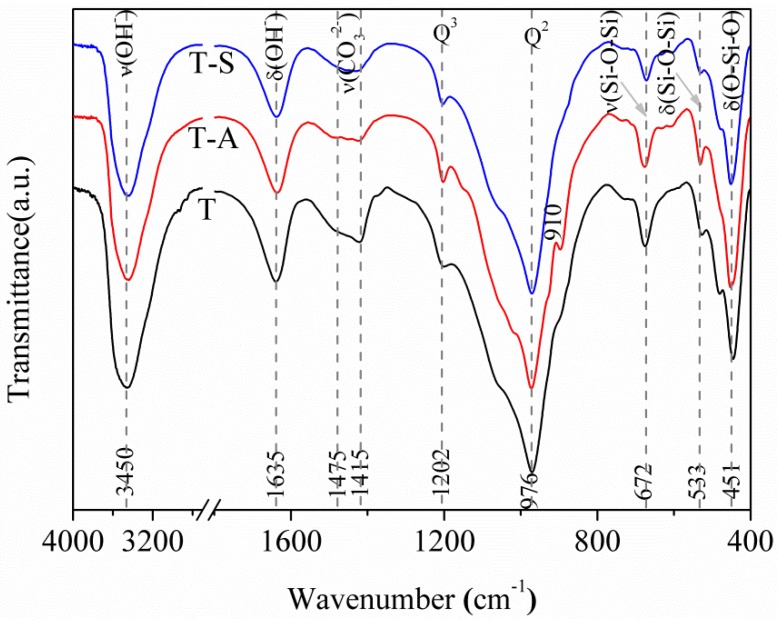
FT-IR spectra of the tobermorite samples.

Figure 3 displays that the resulting three FT-IR spectra plots show similar behaviors concerning the wavenumber from 4000 to 400 cm^−1^ indicating that incorporations of aluminum and sucrose have little effect on the change of silicate chain structure. The FT-IR spectra display a main narrow bands around 976 cm^−1^, typical of the Si-O stretching vibrations generated by Q^2^ silicon sites [2,27,29]. Band at 1202 cm^−1^ is due to Si-O stretching vibrations in Q^3^ silicon sites specific for tobermorite [27]. A shoulder at 910 cm^−1^ for T-A sample may be assigned to O-H bending vibrations in Al-OH-Al bonds (octahedral aluminum) [2]. The band at 672 cm^−1^ is due to Si-O-Si stretching vibrations in SiO_4_ tetrahedra. It should be noted that the higher band intensity of Q^3^ silicon sites alongside the weaker band intensity of SiO_4_ tetrahedra for T-A sample and T-S sample compared to the cases for the control sample indicate an increased polymerization degree of silicate chains by incorporating aluminum and sucrose. The bands in the range of 533–451 cm^−1^ are assigned to O-Si-O deformation and bending modes of SiO_4_ tetrahedra [2,27,29]. The band at 1635 cm^−1^ is due to H-O-H bending vibrations and the band centered at 3450 cm^−1^ is associated with O-H stretching vibrations [2,25,27,29]. In the region of 1475 to 1415 cm^−1^, the spectra show a very broad band corresponding to asymmetric stretching of CO_3_^2−^.

#### 3.2.3. NMR Analysis

**Figure 4:**
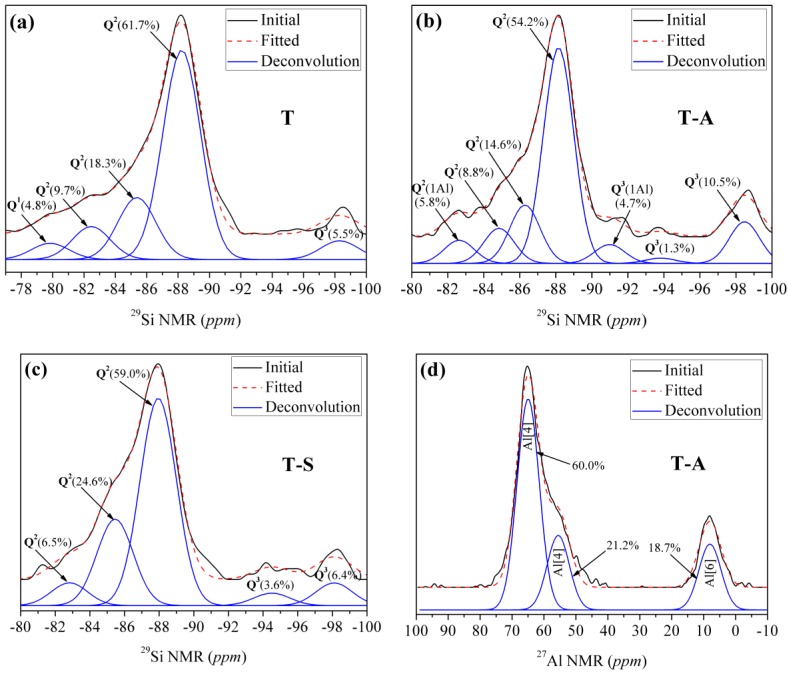
NMR spectra of the tobermorite samples: (**a**) ^29^Si NMR spectra of the control sample; (**b**) ^29^Si NMR spectra of the T-A sample; (**c**) ^29^Si NMR spectra of the T-S sample; (**d**) ^27^Al NMR spectra of the T-A sample.

By means of ^29^Si-NMR investigations, it is possible to detect whether the incorporations of aluminum and sucrose affect the connectivity of the Si-centers of the synthesized tobermorite samples by identifying different Q sites correlating with the linkage of the SiO_4_ tetrahedra. There are five peaks (Figure 4a) in the ^29^Si NMR spectra of the control sample and the chemical shifts are −98.2 ppm, −88.2 ppm, −85.4 ppm, −82.5 ppm and −79.8 ppm, respectively. The signal at −79.8 ppm is attributed to Q^1^ silicon sites (isolated dimers) referred to the previous studies [3,28]. Maeshima et al. [28] presented ^29^Si-NMR data for naturally occurring 11 Å tobermorite with signals for the Q^2^ sites (chain groups) located at ~−80.5 to −85.3 ppm and signals for Q^3^ sites (chain branching) at ~−91.9 to −96.1 ppm. Our Q^2^ signals locate at −88.2 ppm, −85.4 ppm and −82.5 ppm, respectively, and with slight deviations in comparison to results found in the literature. The signal at −98.2 ppm can be assigned to Q^3^ sites.

As shown in Figure 4b,c, Q^1^ signals disappear in the spectra of T-A sample and T-S sample. In both cases, only Q^2^ and Q^3^ signals can be observed, which is in good agreement with the FT-IR results and also correlates with the high crystallinity of the samples observed via XRD measurements and the associated high order of the structure which leads to the formation of Q^3^ sites. After being incorporated with aluminum, resonances slightly deviate to higher frequency for Q^2^(1Al) and Q^3^(1Al) sites relative to Q^2^ and Q^3^ sites, respectively. By comparison, Coleman [11] identified that Q^2^ and Q^2^(1Al) sites located at −86.5 and −83.5 ppm, while Q^3^ and Q^3^(1Al) sites corresponded with signals at −98.4 and −93.5 ppm, respectively. Gabrovŝek et al. [30] assigned signals at −86 and −82.5 ppm to Q^2^ and Q^2^(1Al) sites, those at −96 and −92 ppm were attributed to Q^3^ and Q^3^(1Al) sites. We have measured two signals for Q^3^ sites (Figure 4b) at −98.5 ppm and −93.9 ppm, respectively, in the spectra of T-A sample. The signals at −88.2 ppm, −86.3 ppm and −84.9 ppm can be assigned to Q^2^ sites according the results of Gabrovŝek et al. [30], in which Q^2^ sites for Al-substituted tobermorite located at −85 to −89 ppm. Our Q^3^(1Al) and Q^2^(1Al) sites are located at −91.0 ppm and −82.6 ppm, respectively. The shift into higher frequency is typical for Al-substituted tobermorite and is due to the formation of neighboring atoms [3]. In the spectra of the T-S sample (Figure 4c), there are five peaks, chemical shifts of which are −98.11 ppm, −94.51 ppm, −87.95 ppm, −85.46 ppm and −82.82 ppm, respectively. The signals at −98.11 ppm and −94.51 ppm are attributed to Q^3^ sites [11,28]. The signals at −87.95 ppm, −85.46 ppm and −82.82 ppm are corresponding to Q^2^ sites [11,30].

It is evident that the proportion of Q^3^ and Q^3^(1Al) sites is increased by incorporating aluminum and sucrose, indicating a higher cross-linking degree of the silicate chains of tobermorite. Thus, it can be concluded that the tobermorite has a higher polymerization degree with incorporations of aluminum and sucrose. Especially, the occurrence of Q^3^(1Al) and Q^2^(1Al) sites indicates Al has more opportunity to occupy the bridging position than Si, which may lower the formation energy (Δ*G*) of tobermorite [10]. The role of aluminum in the aluminosilicate chains of tobermorite structure can be further identified by ^27^Al NMR. Generally, the presence of aluminum in C-S-H has three forms, i.e., coordination numbers of 4, 5 and 6 [3,9,31]. Al [4] coordination sites with center bands are at ∼50–70 ppm, Al [5] ∼30–40 ppm, and Al [6] ∼0 ppm, respectively. The ^27^Al NMR spectra for T-A sample, presented in Figure 4d, are predominantly Al [4] with some Al [6]. The two Al [4] resonances at 64.95 and 55.54 ppm correspond well with those reported for Al-substituted 11 Å tobermorite [3,11,32]. The assignments of the two distinct Al [4] have been associated with the isomorphous substitution of Al for Si in two different environments, the 64.95 ppm resonance corresponding to substitution in the middle chain bridging Q^2^ and the 55.54 ppm resonance to substitution in the branching Q^3^ sites [32]. Thus, Al occupancy of Q^2^ bridging and Q^3^ branching sites indicates that Al links silicate chains together, creating silicate units that are connected by bridging and branching Al tetrahedra. The resonance at 8.04 ppm is attributed to Al[6] probably from some calcium aluminate hydrate phase, which includes Al(OH)_6_^3−^ or O*_x_*Al(OH)_6−*x*_^(3+*x*)−^ octahedral [9]. This is consistent with the above FT-IR conclusion about the O-H bending vibrations in Al-OH-Al bonds at 910 cm^−1^.

#### 3.2.4. SEM Analysis

**Figure 5:**
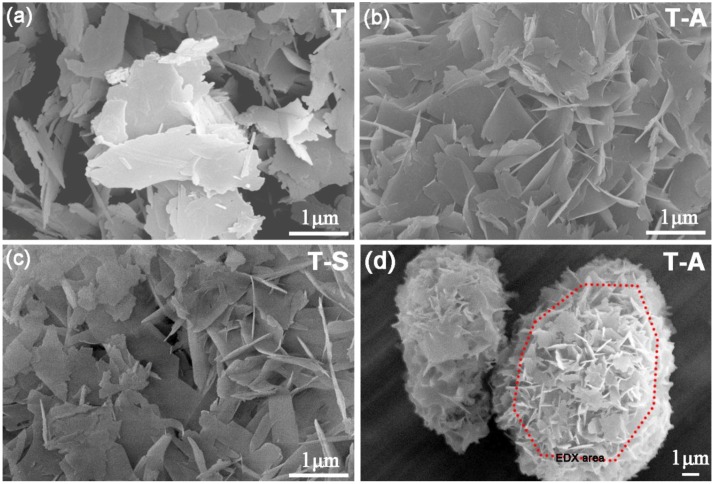
SEM images of the tobermorite samples: (**a**) the control sample; (**b**) and (**d**) the T-A sample; (**c**) the T-S sample.

**Figure 6:**
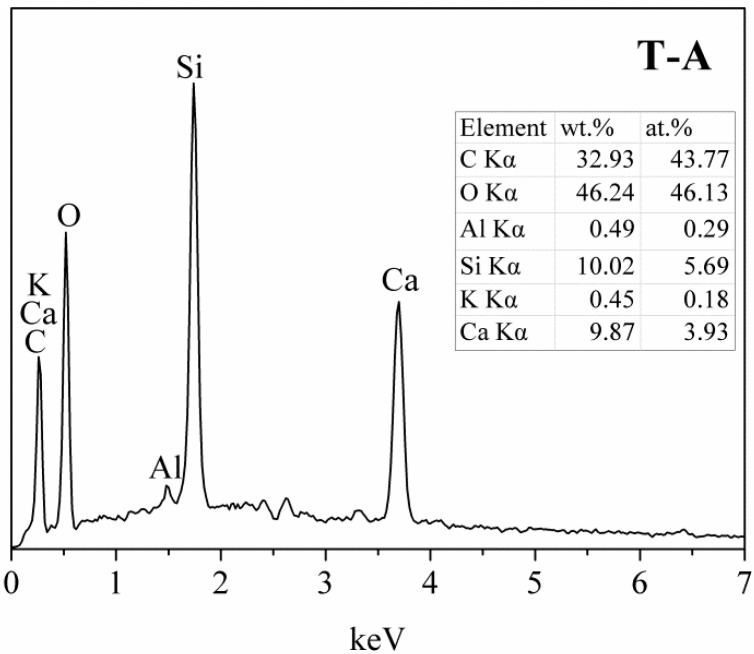
EDX spectra of the T-A sample in Figure 5d.

Micromorphology of the tobermorite samples are displayed in Figure 5. The control sample shows a thin platy morphology (Figure 5a), which is the crystal habit of tobermorite [2], with a size of approximately 1–2 μm. The morphology of tobermorite has almost no change after incorporated with aluminum except for more arranged of foils, as shown in Figure 5b. In the presence of sucrose, some of tobermorite display a lathy morphology with around 1 μm long and 300 nm wide (Figure 5c). It should be noted that in all cases the tobermorite samples present irregular spherical particles with an open framework surface structure (Figure 5d). This porous microstructure may develop from the stacking of tobermorite plates, foils or lathes, and means a large surface area and pore volume to which adsorbates are readily accessible. Figure 6 shows the aluminum-incorporated tobermorite predominantly consists of Ca and Si with trace of Al and K. The Ca/Si molar ratio of this tobermorite is 0.69, which is slightly smaller than the ideal composition of tobermorite (0.8).

#### 3.2.5. BET Analysis

**Figure 7:**
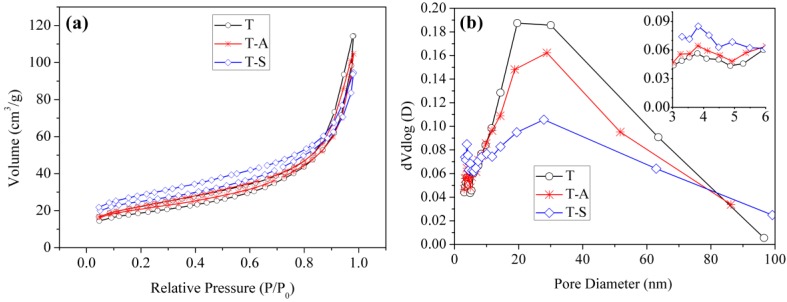
N_2_ adsorption–desorption isotherms (**a**); and pore-size distribution curves (**b**) of the tobermorite samples.

The N_2_ adsorption–desorption isotherms and pore size distributions of the tobermorite samples are shown in Figure 7. All the isotherms are similar and can be classified as type IV [33], which indicates that the framework structure of tobermorite does not change by incorporating aluminum and sucrose. The isotherms show a H4-type hysteresis loop, which is typically observed for aggregates of plate-like particles [33,34]. This result is consistent with the SEM observations (Figure 5). Moreover, the pore size distributions of the tobermorite samples confirm the pore size ranges from 3 to 100 nm and is bimodal with trace of small mesopores (~4 nm) as well as dominantly larger ones (~20 nm). The small mesopores and larger ones probably come from the aggregation of primary particles and secondary particles, respectively. The average pore diameters of T, T-A and T-S samples are 10.56 nm, 8.71 nm and 6.56 nm, respectively. Thus. the pore structure of tobermorite is refined by incorporating aluminum and sucrose. The BET specific surface areas of T, T-A and T-S samples are 67.04 m^2^/g, 74.39 m^2^/g and 89.19 m^2^/g, respectively, which are higher than the previous value for the synthetic tobermorite (57 m^2^/g) [35]. It should be noted that the incorporations of aluminum and sucrose also play a productive role in increasing the porosity of tobermorite. The larger surface area provides a larger number of adsorption sites, which may be ideally effective for pollutant adsorption when it is directly used as an adsorbent.

### 3.3. Cr(VI) Adsorption Characteristics

#### 3.3.1. Effects of Solution pH

**Figure 8:**
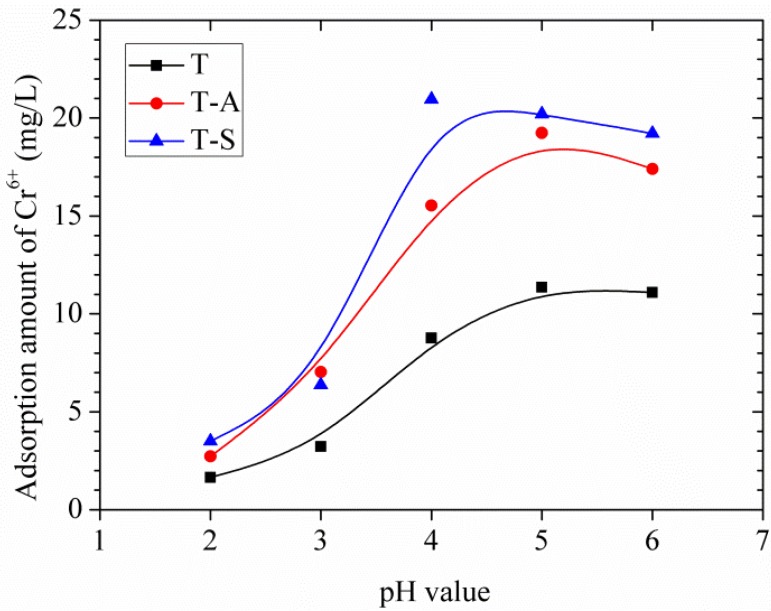
Effects of initial pH on adsorption amount of Cr(VI).

The adsorption of Cr(VI) from aqueous solutions is highly dependent on the pH of the solution because the pH affects not only the surface properties of the adsorbents but also the ionic state of chromium during the reaction. In this study, the effects of pH on the adsorption amount of the tobermorite samples are shown in Figure 8. The tendencies of adsorption amount with the increase of pH are quite similar for different tobermorite samples. The adsorption amount increases when pH increases from 2.0 to 4.0, and the maximum adsorption amount of Cr(VI) by T-S sample is found at about pH 4.0 while those of T-A and T samples are at about pH 5.0. Moreover, the adsorption amount of Cr(VI) by all samples slightly decreases as pH increases to 6.0. This result is consistent with the previous results [15,16] and can be explained as follows: different species of Cr(VI) (e.g., Cr_2_O_7_^2−^, CrO_4_^2−^, and HCrO_4_^−^) coexist at acidic condition. The predominant Cr(VI) species at pH 2.0–4.0 is the monovalent HCrO_4_^−^, which is favorable adsorbed since it has a low adsorption free energy [19]. On the other hand, when the pH value is low, adsorbent (tobermorite samples) static charges are presented in positively charged form, so the adsorption rate of Cr(VI) is relatively high by electrostatic interaction. Apart from the electrostatic interaction effect, complexation of HCrO_4_^−^ with the interlayer or Ca^2+^ structural calcium in the structure of tobermorite may occur during the absorption process. The absorption mechanism will be interpreted in detail in Section 3.3.4. The decrease of adsorption amount at pH 6.0 may be due to: (1) more adsorption sites required for the bivalent Cr_2_O_7_^2−^and CrO_4_^2−^, which are the predominant species at pH 6.0; (2) competitions from increasing OH^−^ ions with Cr(VI) anions for the adsorption sites; and (3) more negative charges on the surface of tobermorite [15,16]. In comparison, the adsorption amounts of Cr(VI) by T-S and T-A samples are higher than that of T sample at the same pH value. This may be because the amount of adsorption sites in tobermorite incorporating aluminum and sucrose is substantially more than those in the control sample.

#### 3.3.2. Effects of Contact Time and Adsorption Kinetic Study

**Figure 9:**
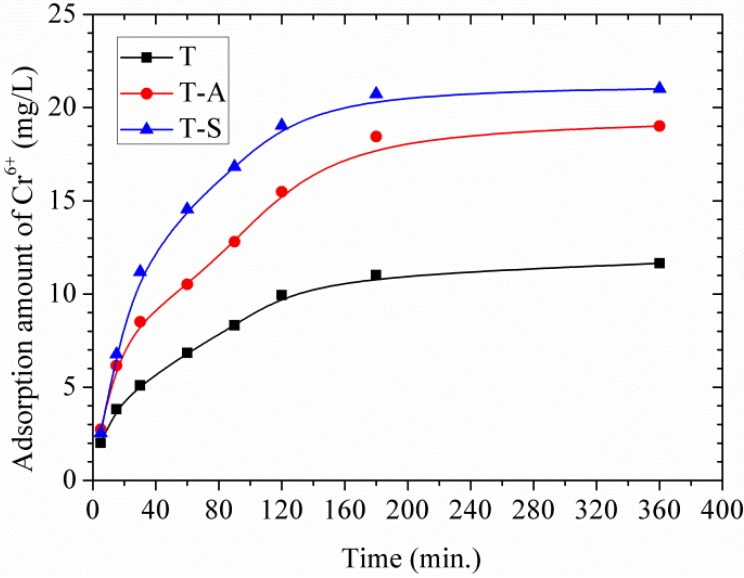
Effects of contact time on adsorption amount of Cr(VI).

Figure 9 displays the adsorption amount of Cr(VI) by tobermorite as a function of time. All the materials show fast adsorption during the first 60 min of the reaction and a much slower subsequent removal followed by an equilibrium state within 3 h. This equilibrium time is close to those of magnetite nanoparticles (2 h) [19], natural clay (2–3 h) [14,15,16] fly ash (3 h) [17] and is much higher than that of activated carbon (24 h) [18]. It should be noted that the adsorption of Cr(VI) onto tobermorite is a combination of physical adsorption and chemical adsorption since the physical adsorption is usually a rapid adsorption process [15,16].

To investigate the rate-controlling mechanism of the adsorption process, adsorption kinetic was modeled by the pseudo-first-order and pseudo-second-order models, which can be expressed as follows [15,16]:

(1) $$\mathrm{pseudo-first-order}:\;\ln\left(q_{e}-q_{t}\right)=\ln q_{e}-k_{1}\cdot \;t,$$

(2) $$\mathrm{pseudo-second-order}:\;\frac{t}{q_{t}}=\frac{1}{k_{2}\cdot q_{e}^{2}}+\frac{1}{q_{e}}\cdot \;t,$$

where *q_t_* (mg/g) and *q_e_* (mg/g) are the adsorption amount at time *t* (min) and equilibrium, respectively. *k*_1_ (min^−1^) and *k*_2_ (mg/g/min) are the pseudo-first-order and pseudo-second-order rate constants of adsorption, respectively.

As listed in Table 2, the adsorption of Cr(VI) follows the pseudo-second-order model better than the pseudo-first-order model. This indicates that the rate-limiting step may be chemical adsorption involving valency changes through sharing or exchange of electrons between tobermorite and Cr(VI) [14,15,16]. Comparing the *q_e_* values for each sample, they follow the order: *q_e_* T-S > *q_e_* T-A > *q_e_* T. The initial adsorption rate (*h*, mg/g/min) can be calculated from *h* = *k*_2_·*q_e_*^2^ and follows the order: *h* T-S > *h* T-A > *h* T, suggesting a more rapid adsorption of Cr(VI) onto the T-S sample at a lower initial concentration.

#### 3.3.3. Effects of the Initial Concentration of Cr(VI) and Adsorption Isotherms

**Figure 10:**
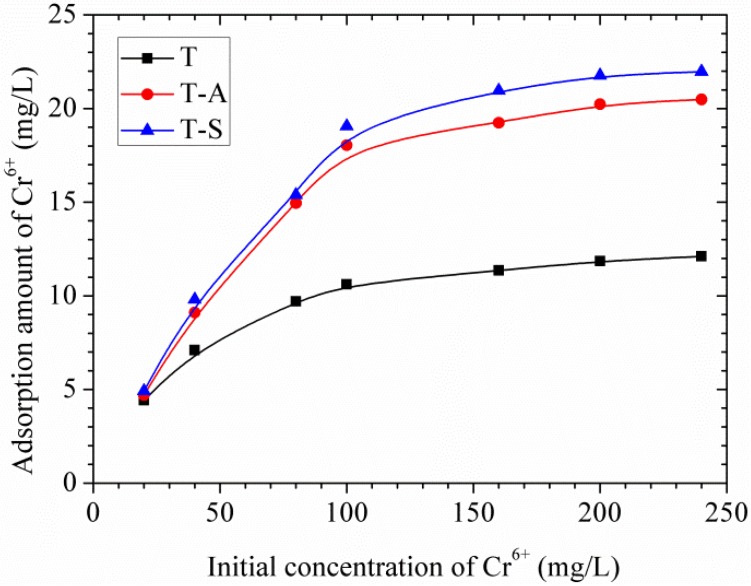
Effects of initial concentration of Cr(VI) on adsorption amount of Cr(VI).

The effects of the initial Cr(VI) concentration on the adsorption amount are shown in Figure 10. It can be seen that the adsorption of Cr(VI) by tobermorite gradually increases as the initial concentration increases until the saturation point is attained at 160 mg/L, therefore a plateau is reached. This may be because available sites for adsorption remain constant for a fixed amount of tobermorite. The experimental data were simulated using the Langmuir and Freundlich models, which can be expressed as follows [15,16]:

(3) $$\mathrm{Langmuir}:\;\frac{C_{e}}{q_{e}}=\left(\frac{1}{k_{L}\cdot q_{max}}\right)+\left(\frac{C_{e}}{q_{max}}\right),$$

(4) $$\mathrm{Freundlich}:\;\ln q_{e}=\ln k_{F}+\frac{1}{n}\cdot \ln C_{e},$$

where *C_e_* is the equilibrium concentration of adsorbate in solution (mg/L). *q_e_* and *q_max_* (mg/g) are the amounts adsorbed at equilibrium and the maximum adsorption capacity for monolayer formation on adsorbent, respectively. *k_L_* (L/mg) is the Langmuir constant related to the maximum adsorption capacity and the energy of adsorption. *k_F_* is the Freundlich constant and *n* is the heterogeneity factor.

The value of theoretical parameters and constants were calculated from the intercept and slope of the linear equation, respectively, and the results are summarized in Table 3. The Langmuir isotherm correlates better than Freundlich due to the higher *R*^2^ values, showing that the adsorption of Cr(VI) on the whole surface of tobermorite samples is uniform. In other words, the whole surface has identical adsorption activity and therefore the adsorbed Cr(VI) ions do not interact or compete with each other, and they are absorbed in the form of an almost complete monolayer coverage on the surface of tobermorite adsorbent [15,16]. The Langmuir maximum adsorption amount for Cr(VI) is in the order: T-S (31.65 mg/g) > T-A (28.92 mg/g) > T (14.27 mg/g). The incorporations of aluminum and sucrose promote the formation and crystallinity of tobermorite during hydrothermal synthesis. On the other hand, the surface area of tobermorite is significantly increased by sucrose, resulting in more adsorption sites for Cr(VI). More “empty” positions exist needing for charge balance in the Al-substituted tobermorite [10]. All the modifications make the tobermorite samples have enhanced adsorption capacity. In the case of Langmuir isotherm the dimensionless coefficient *K_L_* values lie between 0 and 1 showing favorable adsorption. Moreover, the values of *n* in the Freundlich isotherm are in the range of 1–10, representing a good adsorption [15,16]. In this work, the exponent of *n* is 1.67–2.54 (1 < *n* < 10), which reflects that the adsorption of Cr(VI) onto tobermorite is favorable.

Table 4 compares the maximum adsorption capacities of Cr(VI) by tobermorite obtained from the Langmuir model in the present work with those reported in literatures. It can be seen that the adsorption capacities T-A sample and T-S samples are not the highest among the materials in literatures, but are higher than those of zeolite and clay, which have been used as adsorbent for years owing to their high cation exchange ability and high affinity for heavy metals [14,15,16]. The high adsorption ability of the tobermorite adsorbent can be ascribed to several factors such as the mesoporous structure, high specific surface area and intrinsic chemical nature of tobermorite. The adsorption mechanism will be discussed in detail below. In other words, it can be predicted that the aluminum- and sucrose-incorporated tobermorites prove to have promising potential for the treatment of Cr(VI) waste water applicable in industry, agriculture, livelihood and other fields in large scale for its low toxicity and cost and high efficiency in the future.

#### 3.3.4. Adsorption Mechanism

**Figure 11:**
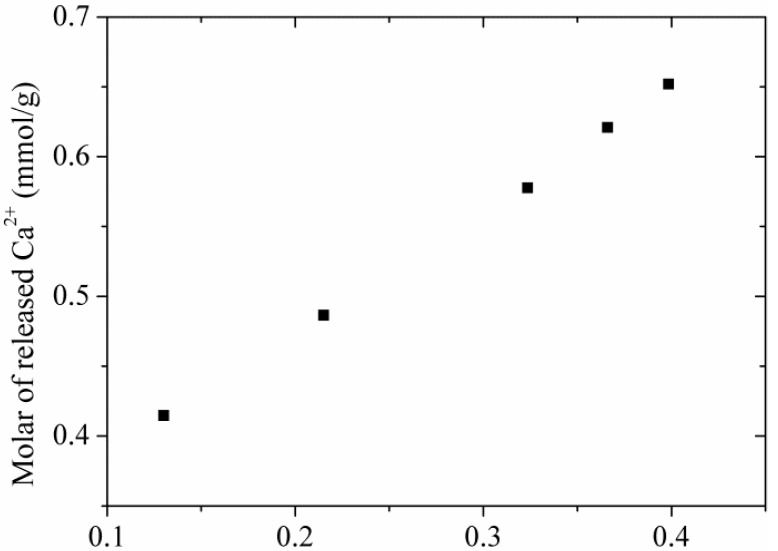
The amounts of Ca^2+^ released from tobermorite at different adsorption amounts of Cr(VI).

**Figure 12:**
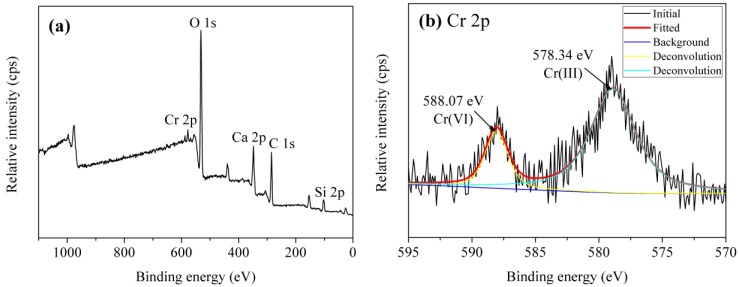
XPS spectra of the tobermorite sample after absorption: (**a**) survey; and (**b**) Cr 2p.

The adsorption of Cr(VI) by tobermorite is a complex process, to better understand the adsorption mechanism, the concentrations of Ca^2+^ were analyzed. Figure 11 displays a nearly linear relationship of the molar amount of absorbed Cr(VI) and the molar amount of Ca^2+^ in the solution. To better understand the adsorption mechanism, XPS measurement was carried out as shown in Figure 12. It can be seen that the Cr 2p1/2 and Cr 2p3/2 photoelectron peaks are located at 588.07 and 578.34 eV, respectively [36], which are assigned to the characteristic peaks of Cr(VI) and Cr(III), respectively. The results indicate that both Cr(VI) and Cr(III) are present on surface of tobermorite samples during the adsorption process. Cr(VI) may be reduced to Cr(III) in the presence of organic matter [36,37] or at a pH of 2.0–6.0 in an acid environment through following redox reaction:

HCrO_4_^−^ + 7H^+^ + 3e^−^ → Cr^3+^ + 4H_2_O

Then, the Cr(III) cations are absorbed by cation-exchange reaction with Ca^2+^ in the structure of tobermorite. While, the exchange seem be non-stoichiometric, because the molar amount of Ca^2+^ in the solution after absorption is always higher than the stoichiometrically calculated molar amount of Cr^3+^. Therefore, the leaching of Ca^2+^ during the reaction process may be due to partial exchange with Cr(VI) and partial hydrolysis of tobermorite in the acidic solution. It is well known that the structure of tobermorite consists of double central layers of CaO octahedra between single silicate chains [5]. The prominent structure feature of tobermorite is the cavity between two adjacent building layers stacked along *c*, the “interlayer space”, which may contain water and Ca^2+^ ions. Thus, the exchange reaction probably takes place from the interlayer Ca^2+^ and certainly can occur on the planar surface or edges of tobermorite derived by the non-equilibrium chemical bonds. At the same time, since all oxygen atoms are shared with Si-O chains in the central layer structure of CaO octahedra in tobermorite, the Ca-O interaction is relatively weaker and easy to break attributed to the acidic nature of the initial solution, accordingly, extra Ca^2+^ ions are released in the solutions. As a result, the structure of tobermorite should be destroyed to some extent, then the reaction of Cr(VI) leads to a partial loss of crystallinity of tobermorite as can be deduced from the decrease of intensities of diffraction peaks in the XRD pattern and the infrared spectra as well as from the morphology observed by SEM.

**Figure 13:**
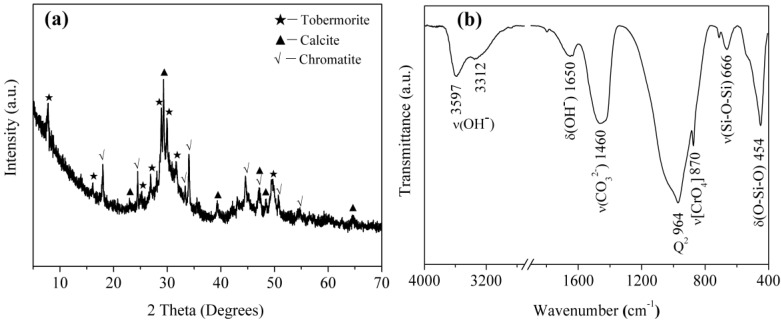
XRD pattern (**a**); and FT-IR spectra (**b**) of the tobermorite reacted with Cr(VI).

It can be seen from Figure 13a that the relative intensities of diffraction peaks from the T-A sample reacted with Cr(VI) solution (adsorption conditions: initial concentration of 160 mg/L; pH of 4.0; contact time of 6 h) at 7.8° (2θ), 28.9° (2θ), 29.9° (2θ) and 31.8° (2θ) decrease with respect to the unreacted sample (Figure 1). This is also reflected in the infrared spectra (Figure 13b). The band attributed to Q^3^ silicon sites disappears indicating a decreased polymerization degree of silicate chains. In addition, The reaction of tobermorite with Cr(VI) leads to precipitation of chromatite (JCPDS card No. 75-0936, Ca(CrO_4_)) as shown in the corresponding XRD pattern (Figure 13a). Infrared analyses show the presence of a single, characteristic, sharp band at 870 cm^−1^ (Figure 13b), assigned to the stretching vibrations of the chromate [CrO_4_] group [38]. The sharp band at 964 cm^−1^ is due to the Si-O stretching vibrations generated by Q^2^ silicon sites. The band at 666 cm^−1^ is due to Si-O-Si stretching vibrations in SiO_4_ tetrahedra and the band at 454 cm^−1^ is assigned to the bending modes of SiO_4_ tetrahedra. Abroad band centered at 1460 cm^−1^ corresponds to the asymmetric stretching of CO_3_^2−^. The band at 1650 cm^−1^ is due to H-O-H bending vibrations. In the O-H stretching region, the reacted tobermorite yields two bands centered at 3597 and 3312 cm^−1^. The first band is associated with vibrations involving less hydrogen-bonded protons in water molecules and the second one is probably due to O-H stretching of more strongly hydrogen-bonded water molecules in the interlayer of tobermorite [2,27,29]. 

**Figure 14:**
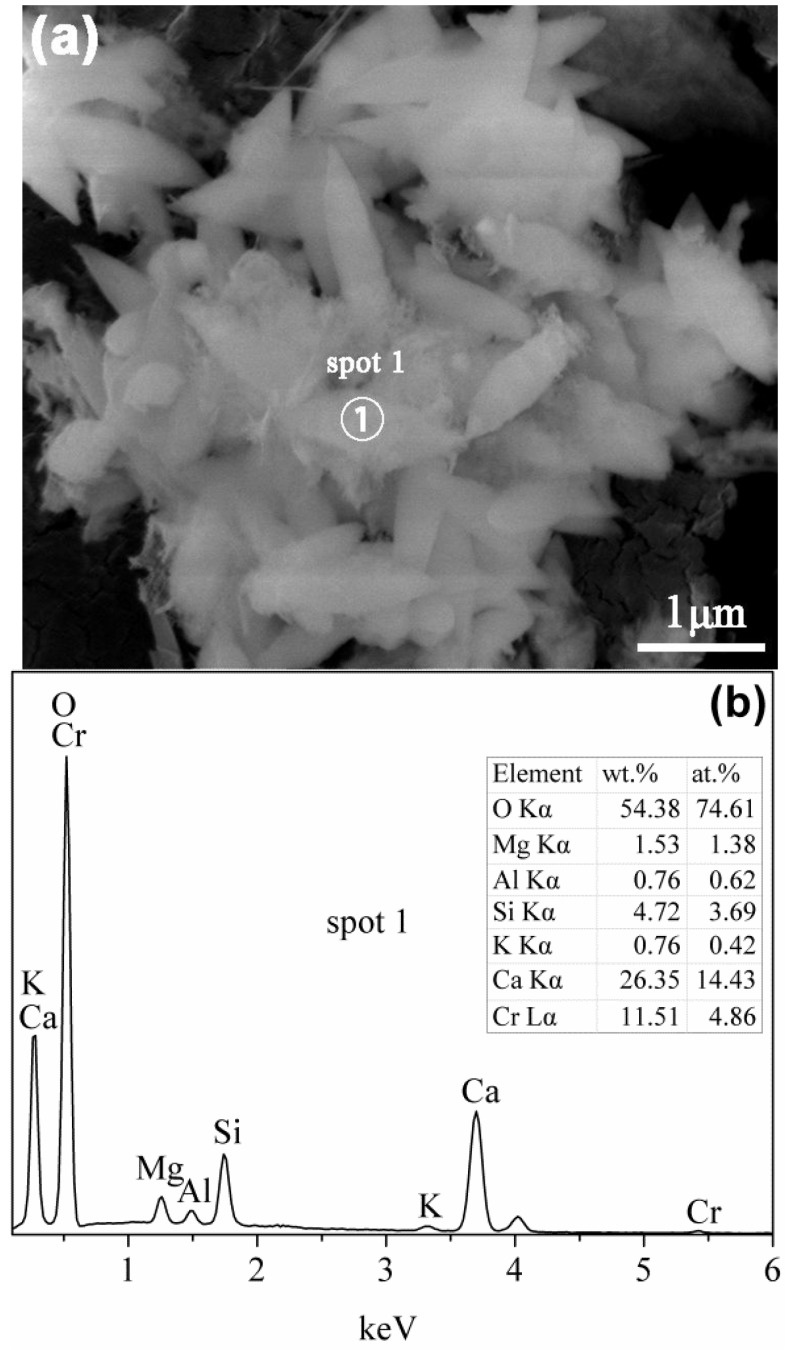
SEM image (**a**); and EDX spectra (**b**) of the tobermorite reacted with Cr(VI).

SEM image illustrates that the tobermorite reacted with Cr(VI) shows a leaf-like morphology with a size of around 1 μm long and 300 nm wide (Figure 14a). It is noteworthy that this tobermorite seems to be smaller than the unreacted sample (Figure 5) and has a sharp tip. EDX spectra of the reacted tobermorite demonstrate the existence of Ca and Si as well as traces of Cr, K, Mg, and Al (Figure 14b). The detected Si peak is significantly less intense than the Ca peak by more than 50% and also less intense than the unreacted sample (Figure 6). These results imply partial hydrolysis of tobermorite and support the XRD and IR data. Higher intensity of Ca peak may be due to the precipitation of chromatite, which is difficult to distinguish among the tobermorite crystals.

**Figure 15:**
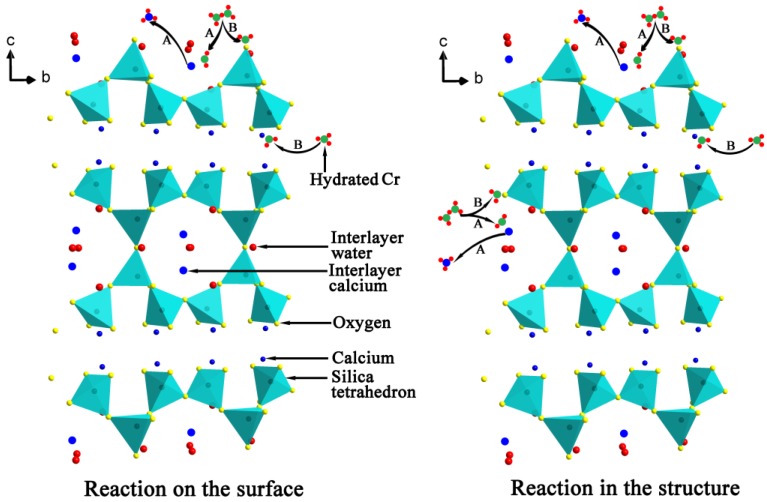
Schematic presentation of the adsorption mechanism of Cr(VI) by tobermorite: (**A**) ion exchange reaction; and (**B**) immobilization effect.

The above analyses indicate that the adsorption of Cr(VI) onto tobermorite is driven by multiple mechanisms, including physisorption and chemisorption. Undoubtedly, the high surface area arising from the porous structure of tobermorite contributes to the adsorption performance via electrostatic interaction, but the intrinsic nature of tobermorite sometimes is more crucial for the adsorption capacity. The adsorption process of Cr(VI)may arise as a result of the following steps: (1) exchange with Ca^2+^ released from the planar surface or edges of tobermorite crystals, at the same time, partial Cr(VI) may be immobilized though complexation reaction with oxygen linking the building layers; and (2) exchange with the interlayer Ca^2+^ for charge balance and finally with the structural calcium. This adsorption mechanism of Cr(VI) by tobermorite is illustrated in Figure 15. The exchange reaction leads to a loss of crystallinity of tobermorite but not the only reason, because tobermorite inevitably decomposes due to the acidic nature of the initial solution. On the other hand, the reaction of tobermorite with Cr(VI) leads to precipitation of chromatite, which may hinder the exchange process and hence promote the adsorption to achieve equilibrium. The incorporations of aluminum and sucrose increase the crystallinity and yield of tobermorite. It is surprising that the surface area is significantly increased by sucrose, which means more active sites for mass transfer and more broken bonds. Due to Al substitution for Si, more “empty” positions exist needing for charge balance and the (Al)Si-O-Ca linkages become more easy to break because of lower Al-O binding energy [10]. Thus, the aluminum- and sucrose-incorporated tobermorites have superb adsorption capacity. 

## 4. Conclusions

In summary, the formation of tobermorite under hydrothermal conditions was modified by incorporating aluminum and sucrose. It was found that the crystallinity and yield of tobermorite were significantly promoted with aluminum addition of Al/(Al+Si) = 0.03 or at a sucrose concentration of Su/Ca = 0.05. FT-IR and NMR results demonstrate that the polymerization degree of silicate chains was increased under the presence of aluminum and sucrose. SEM observations show tobermorite presented irregular spherical particles with an open framework surface structure to which adsorbates may be readily accessible. In addition, it is surprising that the surface area was significantly increased by sucrose, and the BET specific surface areas of T, T-A and T-S samples were 67.04 m^2^/g, 74.39 m^2^/g and 89.19 m^2^/g, respectively. The adsorption process of Cr(VI) by tobermorite followed the pseudo-second-order model implying a chemical adsorption. Adsorption mechanisms were deduced to be a mixed effect of exchange, immobilization, and precipitation. The adsorption isotherms were well fitted by the Langmuir model which indicates a monolayer adsorption. The maximum adsorption amount for Cr(VI) was in the order: T-S (31.65 mg/g) > T-A (28.92 mg/g) > T (14.27 mg/g) and higher than most of the materials in the literature. Due to superb adsorption capacity, high thermal stability, and low cost for large scale production, this adsorbent has potential for Cr(VI) removal from wastewater.

## Figures and Tables

**Table 1:** Weight change proportion according to Figure 2.

Temperature Range (°C)	Weight Change (%)		
	T	T-A	T-S
40–100	1.48	1.64	1.66
100–400	8.06	8.95	9.48
650–720	0.54	0.13	0.10
Total	12.68	13.12	13.17

**Table 2:** A comparison of pseudo-first-order and pseudo-second-order kinetic model for the Cr(VI) adsorption by tobermorite.

Sample	Pseudo-First-Order Model			Pseudo-Second-Order Model		
	*q_e_* (mg/g)	*k*_1_ (min^−1^)	*R*^2^	*q_e_* (mg/g)	*k*_2_ × 10^3^ (mg/g/min)	*R*^2^
T	10.79	0.01518	0.9851	12.98	1.9040	0.9929
T-A	20.37	0.01757	0.9125	21.48	1.0251	0.9883
T-S	22.57	0.02257	0.9652	23.43	1.2394	0.9967

**Table 3:** Langmuir isotherm model for the Cr(VI) adsorption by the tobermorite samples.

Sample	Langmuir Isotherm			Freundlich Isotherm		
	*q_m_* (mg/g)	*K_L_* (L/mg)	*R*^2^	*k_F_*	*n*	*R*^2^
T	14.27	0.0243	0.9991	1.5362	2.54	0.9293
T-A	28.92	0.0116	0.9768	0.9557	1.70	0.9277
T-S	31.65	0.0109	0.9767	0.9704	1.67	0.9341

**Table 4:** Comparison of adsorption ability of Cr(VI) on various absorbents.

Adsorbents	*S_area_* (m^2^/g)	*q_max_* (mg/g)	Equilibrium Time (h)	Optimum pH	T (°C)	References
T	67.04	14.27	3	4	20	This study
T-A	74.39	28.92	3	4	20	This study
T-S	89.19	31.65	3	3	20	This study
Zeolite-rich tuff	5.29	1.16	2	3	18	[14]
Vermiculite	-	27	3	4	25	[15]
Bentonite	-	24	3	4	25	[15]
Attapulgite	-	15	3	4	25	[15]
Zeolite	-	13	3	4	25	[15]
Natural clay	116	4.5	2	5	20	[16]
Natural clay	140	10.9	2	5	20	[16]
Activated carbons	1462	262	24	2	60	[18]
Magnetite nanoparticles	-	15.3	2	2.5	25	[19]
CNT	700 (SWCNT); 270 (MWCNT)	2.35 (SWCNT); 1.26 (MWCNT)	1	2.5	25	[20]
chitosan	-	215	-	4	25	[21]
